# *Toxoplasma gondii* ROP18 induces maternal–fetal dysfunction by downregulating CD73 expression on decidual macrophages

**DOI:** 10.1186/s13071-025-06713-2

**Published:** 2025-02-24

**Authors:** Jingjing Guo, Xiaohui Wang, Lei Wei, Shuai Li, Junwei Wang, Yan Zhang, Ruohan Yang, Han Zhang, Aiqun Xu, Yuzhu Jiang, Xuemei Hu

**Affiliations:** 1 College of Basic Medicine, Qilu Medical University, Zibo, Shandong, Shandong 255000 People’s Republic of China; 2 Department of Immunology, Binzhou Medical University, Yantai, Shandong 264003 People’s Republic of China; 3 Department of Gynecology and Obstetrics, Yantai Affiliated Hospital of Binzhou Medical University, Yantai, Shandong 264000 People’s Republic of China

**Keywords:** *Toxoplasma gondii*, ROP18, Decidual macrophage, CD73, Abnormal pregnancy outcome

## Abstract

**Background:**

Decidual macrophages (dMφ) are pivotal in maintaining maternal–fetal immune tolerance during normal pregnancy by expressing a range of immune-suppressive molecules, including CD73. It has been demonstrated that *Toxoplasma gondii* (*T*. *gondii*) infection during pregnancy can impair dMφ function, potentially leading to adverse pregnancy outcomes, through downregulation of these inhibitory molecules. *T*. *gondii* rhoptry protein 18 (*Tg*ROP18), a key virulence factor of *T*. *gondii*, is associated with the incapacitation of the host’s innate and adaptive immune responses to protect the parasite from elimination. However, the role of *Tg*ROP18 in modulating CD73 expression on dMφ and the underlying mechanisms remain to be elucidated.

**Methods:**

Wild-type (WT) and CD73-deficient (CD73^−/−^) pregnant mice were subjected to intraperitoneal injection of *T*. *gondii* RH or RH-Δ*rop18* on gestational day (Gd) 8, and subsequently euthanized on Gd 14. Pregnancy outcomes were then evaluated, and the expression levels of CD73, arginase 1 (Arg-1), and interleukin 10 (IL-10) were quantified by flow cytometry. Mononuclear cells isolated from the human aborted decidual tissues were also infected with *T*. *gondii* RH or RH-Δ*rop18* for the analysis of CD73 expression with flow cytometry. Additionally, infected human dMφ were used to assess the expression levels of CD73, Arg-1, IL-10, and their associated signaling molecules by western blot analysis. Furthermore, chromatin immunoprecipitation (ChIP) assays were performed to validate the involved signaling pathways.

**Results:**

Compared with the *T*. *gondii* RH-infected group, milder adverse pregnancy outcomes and attenuated expression levels of CD73 on dMφ were observed in *T*. *gondii* RH-Δ*rop18*-infected pregnant mice and human decidual tissues. Lysine-specific histone demethylase1 (LSD1) and snail family transcriptional repressor 1 (SNAIL1) were found to be involved in the downregulation of CD73 expression on dMφ following *T*. *gondii* infection. Subsequently, reduced expression of CD73 contribute to the downregulation of Arg-1 and IL-10 expression through adenosine A2a receptor (A2AR) / protein kinase A (PKA) / phosphorylated cAMP-response element binding protein (p-CREB) / CCAAT enhancer binding protein B (C/EBPβ) pathway.

**Conclusions:**

*Tg*ROP18 can significantly reduce CD73 expression on dMφ through LSD1/SNAIL1 pathway, subsequently leading to the decreased expression levels of Arg-1 and IL-10 via adenosine/A2AR/PKA/p-CREB/C/EBPβ pathway, which ultimately contributes to maternal–fetal tolerance dysfunction of dMφ.

**Graphical Abstract:**

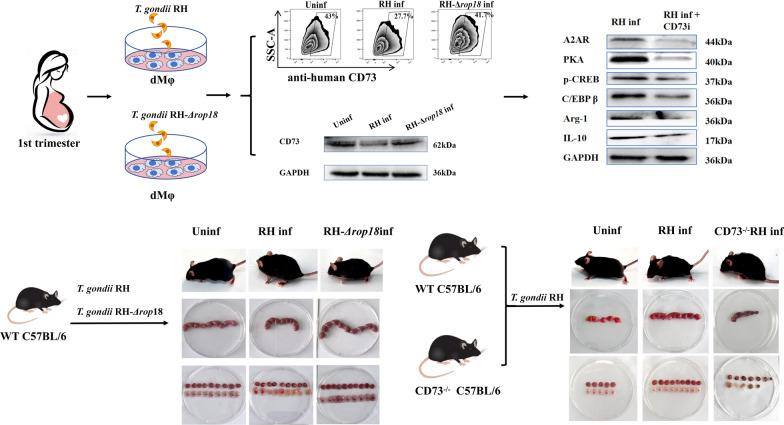

## Background

*Toxoplasma gondii* is an obligate intracellular protozoan parasite affecting nearly one-third of the world population [1]. Once infection occurred during pregnancy, *T*. *gondii* can be transmitted vertically to the fetus, leading to severe complications, such as miscarriage, mental retardation, and congenital malformations [2]. The immune microenvironment at the maternal–fetal interface, primarily composed of various decidual immune cells, immune molecules, and corresponding cytokines, is crucial for maintaining normal pregnancy [3–5]. It has been suggested that the abnormal pregnancy outcomes induced by *T*. *gondii* infection are closely linked to the disruption of the immune microenvironment at the maternal–fetal interface [6, 7]. dMφ represents the second most abundant immune cells at the maternal–fetal interface, comprising 20–25% of decidual immune cells [8]. Our previous studies showed that *T*. *gondii* infection downregulated the expression of immune inhibitory molecules, such as Tim-3 and LILRB4, leading to dMφ dysfunction [9–11].

CD73, also referred to as 5′-nucleotidase, is a surface enzyme widely expressed in various cell types, including immune and placental cells [12, 13]. Studies have shown that patients with unexplained recurrent spontaneous abortion (URSA) exhibit significantly reduced CD73 expression level in decidual tissue, ultimately leading to miscarriage [14]. However, whether the expression level of CD73 on dMφ is altered in response to *T*. *gondii* infection and whether such alterations are associated with adverse pregnancy outcomes require further investigation. Increasing evidences suggest that *T*. *gondii*-derived virulence factors are involved in host interaction, cellular invasion, and immune response [15, 16]. One such high virulence factor is *Tg*ROP18, a member of the rhoptry protein 2 family, could directly phosphorylate and inactivate immunity-related GTPases (IRGs), thus avoiding their accumulation on the *T*. *gondii* parasitophorous vacuole membrane (PVM) and *T*. *gondii*’s clearance in the macrophages [17–19]. Furthermore, *Tg*ROP18 may skew T cell differentiation toward a Th2 phenotype, which facilitates the long-term survival of *T*. *gondii* within the host [20]. However, whether *Tg*ROP18 induces change in CD73 expression on dMφ and the underlying mechanism requires further investigation.

The results of our previous studies have demonstrated that the decreased expression of functional molecules, such as Arg-1 and IL-10, in dMφ following *T*. *gondii* infection could impair the tolerance of dMφ [9–11]. Therefore, further investigation is necessary to determine whether the change of CD73 expression on dMφ induced by *Tg*ROP18 could further influence the expression of Arg-1 and IL-10, which disrupts maternal–fetal tolerance.

In this study, human dMφ and CD73 knockout (CD73^−/−^) pregnant mice were utilized to explore *Tg*ROP18–induced changes in CD73 expression on dMφ during *T*. *gondii* infection and elucidate the molecular mechanisms underlying the immunosuppressive dysfunction of dMφ.

## Methods

### Preparation of *T*. *gondii* tachyzoites

*T*. *gondii* tachyzoites (RH strain) and a ROP18 knockout RH strain (RH-Δ*rop18*) were used in this study. Tachyzoites of the *T*. *gondii* RH strain were inoculated into human foreskin fibroblast (HFF) cells cultured in Dulbecco’s Modified Eagle Medium (DMEM) supplemented with 10% fetal bovine serum (FBS) and penicillin (100 IU/mL) /streptomycin (100 μg/mL). *T*. *gondii* RH-Δ*rop18* was obtained from the department of pathogen biology, Anhui Medical University, and similarly cultured in HFF cells.

### Experimental animals and *T*. *gondii* infection

Experimental animals included wild-type (WT) and CD73-deficient (CD73^−/−^) C57BL/6 mice. WT mice were purchased from Pengyue Laboratory Animal Technology Co., Ltd. (Jinan, China), with certificates of quality assurance. CD73^−/−^ homozygous mice were obtained from Cyagen Biosciences Inc. (Suzhou, China) and bred to obtain more homozygous mice. All mice were housed using an individually ventilated cage (IVC) system in a temperature- and humidity-controlled specific pathogen-free (SPF)-grade animal facility. They were provided with drinking water sterilized using high-pressure steam and SPF-grade food (Jiangsu Biological Engineering Co., Ltd., China).

Mice aged 8–10 weeks were randomly paired at a ratio of 1:2 (male: female). Females with a vaginal plug were recorded as gestational day (Gd) 0 of pregnancy following overnight cohabitation with males. Pregnant mice were intraperitoneally injected with 300 tachyzoites of *T*. *gondii* RH or RH-Δ*rop18* in 200 μL sterile phosphate-buffered saline (PBS) on Gd 8. The uninfected group received an intraperitoneal injection of 200 μL sterile PBS [5]. After fetuses, uteri, and placentas were harvested on Gd 14 for analysis, the number of dMφ and expression level of CD73 were determined. All experimental protocols followed the relevant ethical regulations for animal testing and research, and animal use adhered to Chinese animal care guidelines. Our study was approved by the Institutional Animal Care and Research Advisory Committee at Binzhou Medical University, China (License number: 2017–009-09).

### ***Genotyping of CD73***^***−/−***^*** mice***

Genomic DNA was extracted from mouse tails, and complementary DNA (cDNA) was synthesized using polymerase chain reaction (PCR). The PCR protocol included initial denaturation at 95 °C for 30 s, followed by 40 cycles of denaturation at 95 °C for 10 s, annealing at 65 °C for 30 s, and extension at 72 °C for 5 s, with a final extension at 72 °C for 3 min. PCR products were separated on gels and stained with GelStain (Transgene S.A., Illkirch-Grafenstaden, France) to visualize DNA. Primers for PCR amplification were P1 (5′-GCCAACTTTGTTCATTGGGCTG-3′), P2 (5′-GGAGTGGTTGTAACAGGAGGG-3′), and P3 (5′-GATCTCCAGACAAAGGTCAGCC-3′). Expected PCR product sizes were 410 bp (homozygous), 410 and 620 bp (heterozygous), and 620 bp (WT).

### Cell preparation from mice

Cervical dislocation was used to euthanize pregnant mice at Gd 14. Uteri and placentas were washed two to three times with cold PBS, dissected into 1–3 mm^3^ pieces on ice using ophthalmic scissors, and then digested in 1640 culture medium containing 0.1% collagenase type IV (Sigma-Aldrich, St. Louis, MO, USA) and 25 IU/mL of DNase I (Sigma-Aldrich) for 1 h at 37 °C with shaking. The digested mixture was filtered through a 48-μm sterile mesh, and the resulting cell suspension was centrifuged at 2000 rpm for 10 min. The supernatant was discarded, and the pellets were suspended in PBS solution. Following careful mixing, 3 mL of the cell suspension was gently layered along the tube wall into a 15 mL centrifuge tube containing 3 mL of mouse lymphocyte separation solution (TBD Science, Tianjin, China). The mixture was centrifuged at room temperature at 2,000 rpm for 20 min. Mononuclear cells from the mouse decidual tissue were harvested from the white film layer and resuspended in sterile PBS.

### Collection of human decidual samples

Clinical samples of decidual tissues were obtained from healthy pregnant women undergoing voluntary abortion during their first trimester. Samples were collected at Yantai Affiliated Hospital of Binzhou Medical University and Yantai Maternal and Child Health Hospital. They were preserved in DMEM supplemented with high-glucose (HyClone, GLogan, UT, USA), 100 IU/mL of penicillin, and 100 μg/mL of streptomycin (Sigma-Aldrich) and sent to the laboratory within 2 h. All participants signed a written informed consent before specimen collection and our sample collection procedure was approved by the ethics committee of Binzhou Medical University (approval no.: 2017–016-01).

### Isolation and infection of human decidual mononuclear cells

The decidual tissues were washed with sterile PBS, cut into small pieces using ophthalmic scissors, and then digested with 0.1% collagenase IV (Sigma-Aldrich) and 25 IU/mL of DNase I (Sigma-Aldrich) in a 37 °C incubator for 60 min. The resulting suspension was filtered through a 48-µm mesh and washed twice with sterile PBS. Decidual mononuclear cells were isolated from the white film layer after Ficoll density gradient centrifugation using a human lymphocyte separation medium (TBD Science). These cells were then divided equally into uninfected, RH-infected, and RH-rop18^−/−^-infected groups. In the infected groups, the corresponding *T*. *gondii* tachyzoites were added to the cells at a ratio of 1:3 (*T*. *gondii* tachyzoites: cells) [6]. All cells were cultured in RPMI 1640 medium supplemented with 10% FBS (Gibco, Thermo Fisher Scientific, Waltham, MA, USA), 100 μg/mL of streptomycin, and 100 IU/mL of penicillin for 20 h at 37 °C in a humidified 5% CO_2_ incubator. Afterward, the cells were prepared for flow cytometry staining and analysis.

### Isolation and purification of human dMφ

The decidual mononuclear cells were cultured at 37 °C for 1 h, and cells that adhered to the surface were collected. dMφ were purified using a human CD14 positive isolation kit following the manufacturer’s instructions. Subsequently, they were divided equally into uninfected, RH-infected, and RH-rop18^−/−^-infected groups and then analyzed using western blot analysis.

### M2 macrophage differentiation of THP-1 cells

THP-1 cells were added to 100 mm cell culture dishes (8 × 10^6^ cells per dish) and treated with 50 nM of phorbol 12-myristate 13-acetate (PMA) to induce differentiation into macrophages. After 24 h, the supernatant was discarded, and fresh medium supplemented with 0.1 μM of medroxyprogesterone acetate was added. After 72 h, the cells were polarized into M2 macrophages [21]. Flow cytometry confirmed the M2 phenotype, using antibodies against CD14, CD206, and CD209 for surface staining according to the manufacturer’s instructions. The induced THP-1 cells were then transfected with pcDNA3.1-LSD1 using jetPRIME® reagent (Polyplus, Illkirch, France), infected with *T*. *gondii* RH, and harvested for western blot analysis of SNAIL1 and CD73 expression.

### Quantitative RT-PCR

Total RNA from human dMφ was extracted using TRIzol reagent (Invitrogen, Thermo Fisher Scientific), and cDNA was synthesized with a SuperRT cDNA Synthesis Kit (CoWin BioSciences, Cambridge, MA, USA) according to the manufacturer’s protocol. Messenger RNA (mRNA) expression levels were analyzed through quantitative reverse transcription-PCR (qRT-PCR) using an UltraSYBR One-Step RT-qPCR Kit (CoWin BioSciences) on a Bio-Rad iQ5 multicolor RT-PCR system (Bio-Rad Laboratories, Hercules, CA, USA). Glyceraldehyde 3-phosphate dehydrogenase was used as the normalization control. The experiments were performed in triplicate. The primer sequences for *NT5E* were as follows: Primer 1 forward primer: CCTTTGCAACTTTTCTGTAAGTCTAAA (5′–3′), and reverse primer: TGTCCCTCTTTGAGCACCTG (5′–3′). Primer 2 forward primer: ACTACCAGTTCTTTTACCTGCT (5′–3′), and reverse primer: AAGACCCTTGCTTCTGGGAC (5′–3′). The relative change in CD73 expression was calculated using the 2^−ΔΔCT^method.

### ChIP assay

The SNAIL1 binding regions on the promoters of *NT5E* were predicted using the NCBI JASPAR online database. Primers for chromatin immunoprecipitation (ChIP) -qPCR were synthesized by Sangon Biotech and were listed in quantitative RT-PCR of methods. The ChIP experiment was conducted using the Simple ChIP® Enzymatic Chromatin IP Kit (Cell Signaling, Danvers, MA, USA) following the manufacturer’s instructions. For protein–DNA cross-linking, approximately 2 × 10^7^cells from the uninfected and infected groups were treated with 1% paraformaldehyde at room temperature for 10 min. Glycine was then added to a final concentration of 0.125 M, and the samples were incubated for 5 min at room temperature to stop the cross-linking. After washing twice with ice-cold PBS, the cells were digested to achieve an optimal DNA length of approximately 150–900 bp using lysis buffer and sonication (20% power: 5 s ON/10 s OFF for 5 min on ice). For immunoprecipitation, protein–DNA complexes were incubated with an antibody overnight at 4 °C with shaking. Protein G agarose beads were then added, and the samples were incubated for 2 h at 4 °C with shaking. The beads were washed 4 times with low-salt washing buffer and twice with high-salt washing buffer at 4 °C for 5 min with shaking. DNA was eluted in ChIP elution buffer, reverse cross-linked through incubation with 5 M NaCl and proteinase K for 2 h at 65 °C, and purified using DNA wash buffer. The purified DNA was then analyzed by qPCR using the Ultra SYBR One-Step RT-qPCR Kit (CoWin BioSciences) on a Bio-Rad iQ5 multicolor RT-PCR system (Bio-Rad Laboratories). All experiments were performed in triplicate.

### Flow cytometry staining and analysis

The prepared human and murine decidual mononuclear cell suspensions (1 × 10^6^ cells per group) were first stained for membrane molecules and then for intracellular molecules (Arg-1, IL-10, and C/EBPβ) using a membrane rupture kit according to the manufacturer’s instructions (eBioscience, San Diego, CA, USA). The cells were then analyzed using a BD FACSCanto™ TM II Flow Cytometer (BD Biosciences, Franklin Lakes, NJ, USA) and FlowJo analysis software (FlowJo LLC, Ashland, OR, USA). The gating strategies are provided in Fig. 2.

### Western blot analysis

Purified human dMφ were infected with *T*. *gondii* tachyzoites at a ratio of 1:3 (*T*. *gondii* tachyzoites: cells) with or without 100 μM of CD73i, 10 μM of A2AR inhibitor (A2ARi), or 10 μM of PKA agonist. Approximately 2 × 10^7^ dMφ from each group were cultured in RPMI 1640 medium supplemented with 10% FBS (Gibco, Thermo Fisher Scientific) and penicillin (100 IU/ml) /streptomycin (100 μg/mL) (Sigma-Aldrich) for 20 h at 37 °C in a humidified 5% CO_2_ incubator.

The cultured human dMφ were harvested, lysed with radioimmunoprecipitation assay (RIPA) lysis buffer (Beyotime Biotechnology, Nantong, China) and centrifuged at 12,000 rpm for 20 min at 4 °C. After measuring the protein concentration, equal amounts of protein were loaded onto 10% or 12% sodium dodecyl sulfate–polyacrylamide gel electrophoresis gels and transferred to polyvinylidene fluoride membranes (Millipore Sigma, Burlington, MA, USA). The membranes were blocked at room temperature for 2 h in 5% skim milk in TBS-T buffer, then incubated with primary antibodies overnight at 4 °C on a shaker. They were then incubated with horseradish peroxidase–labeled secondary antibodies (Abmart Inc., Berkeley Heights, NJ, USA) at 37 °C for 1 h. The hybridization signal bands were visualized using an enhanced chemiluminescence detection kit (Yeasen Biotechnology, Shanghai, China). Protein expression levels were determined using ImageJ software. The experiments were replicated three times.

### Statistical analysis

Statistical analyses were conducted using GraphPad Prism 8 software (GraphPad Software, La Jolla, CA, USA). Data are presented as the mean ± standard deviation (SD). Differences between groups were identified using unpaired and paired *t*-tests. One-way analysis of variance was applied with a 95% confidence interval (CI). Statistical significance was set at *P* < 0.05.

## Results

### *T*. *gondii* RH-Δrop18-infected pregnant mice show milder adverse pregnancy outcomes compared with *T*. *gondii* RH-infected mice

**Fig. 1:**
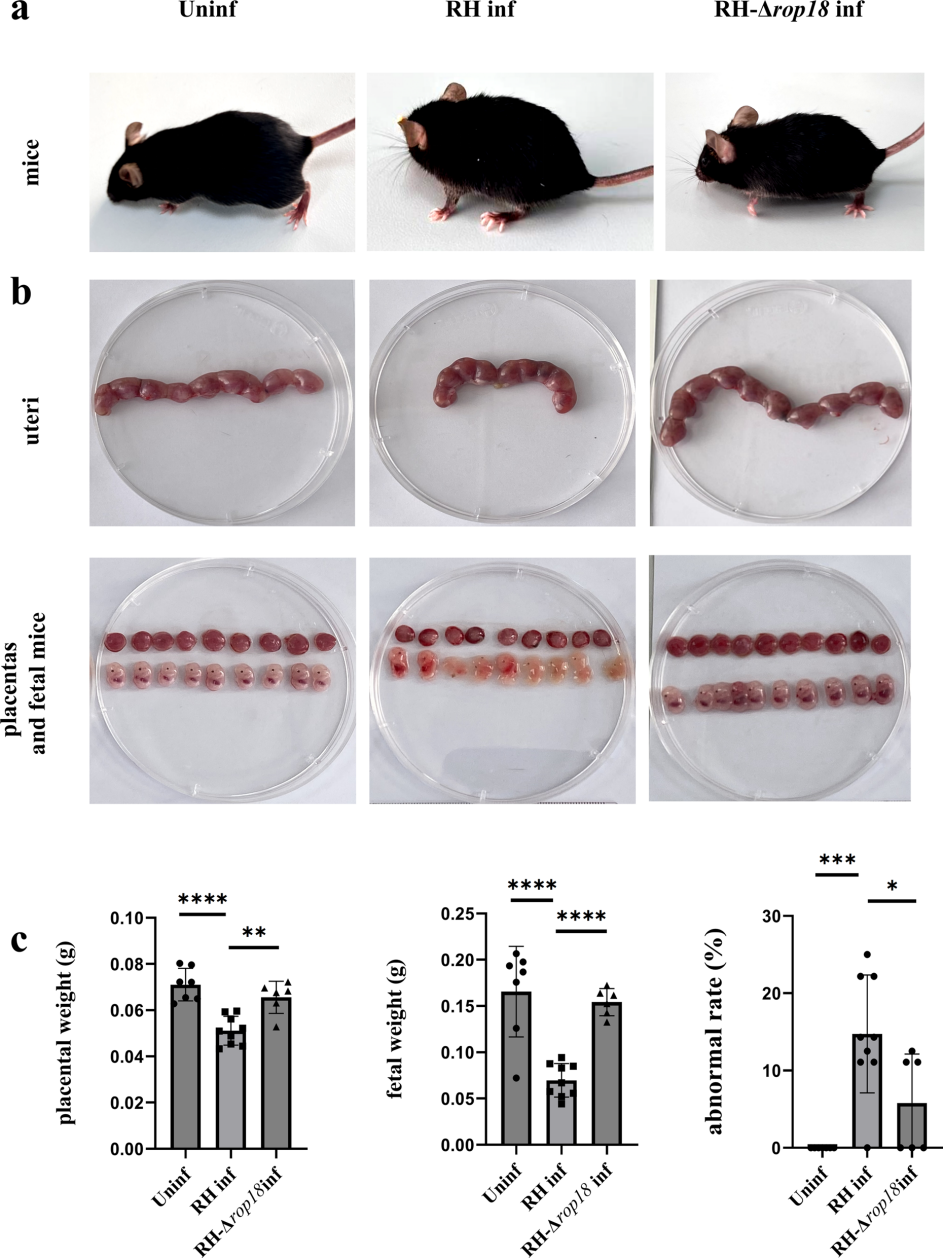
Pregnancy outcomes of the uninfected, RH-infected, and RH-Δ*rop18*-infected mice. (**a**), Pregnant mice in the uninfected, RH-infected, and RH-Δ*rop18*-infected groups. (**b**), Uteri, placentas, and fetuses in the uninfected, RH-infected, and RH-Δ*rop18*-infected groups. (**c)**, Statistical analysis of average placental weight, average fetal weight, and percentage of abnormal fetuses in the uninfected, RH-infected, and RH-Δ*rop18*-infected groups. At least six pregnant mice in each group were identified by unpaired *t*-test. Data are presented as mean ± SD, ^*^P < 0.05, ^**^P < 0.01, ^***^P < 0.001, ^****^P < 0.0001 (unpaired *t*-test)

To determine the effects of *Tg*ROP18 on pregnancy outcomes, pregnant mice were infected with either *T*. *gondii* RH or RH-Δ*rop18*. Observations showed that *T*. *gondii* RH infected mice are obviously slow-moving, with arched backs, raised hair, and drooping tails. *T*. *gondii* RH-Δ*rop18* infected mice move slightly slower, with their backs slightly arched, their fur standing on end, and their tails gently raised (Fig. 1a). In the RH-Δ*rop18*-infected group, placental and fetal development were significantly improved, with increased placental (*P* < 0.01) and fetal weights (*P* < 0.0001) and a decreased rate of abnormal embryos (*P* < 0.05). The rates of fetal death, absorption, and placental necrosis were also reduced compared with those in the RH-infected group (Fig. 1b, c).

### *T*. *gondii* RH-Δrop18 attenuates the reduction in CD73 expression on dMφ induced by *T*. *gondii* RH

**Fig. 2:**
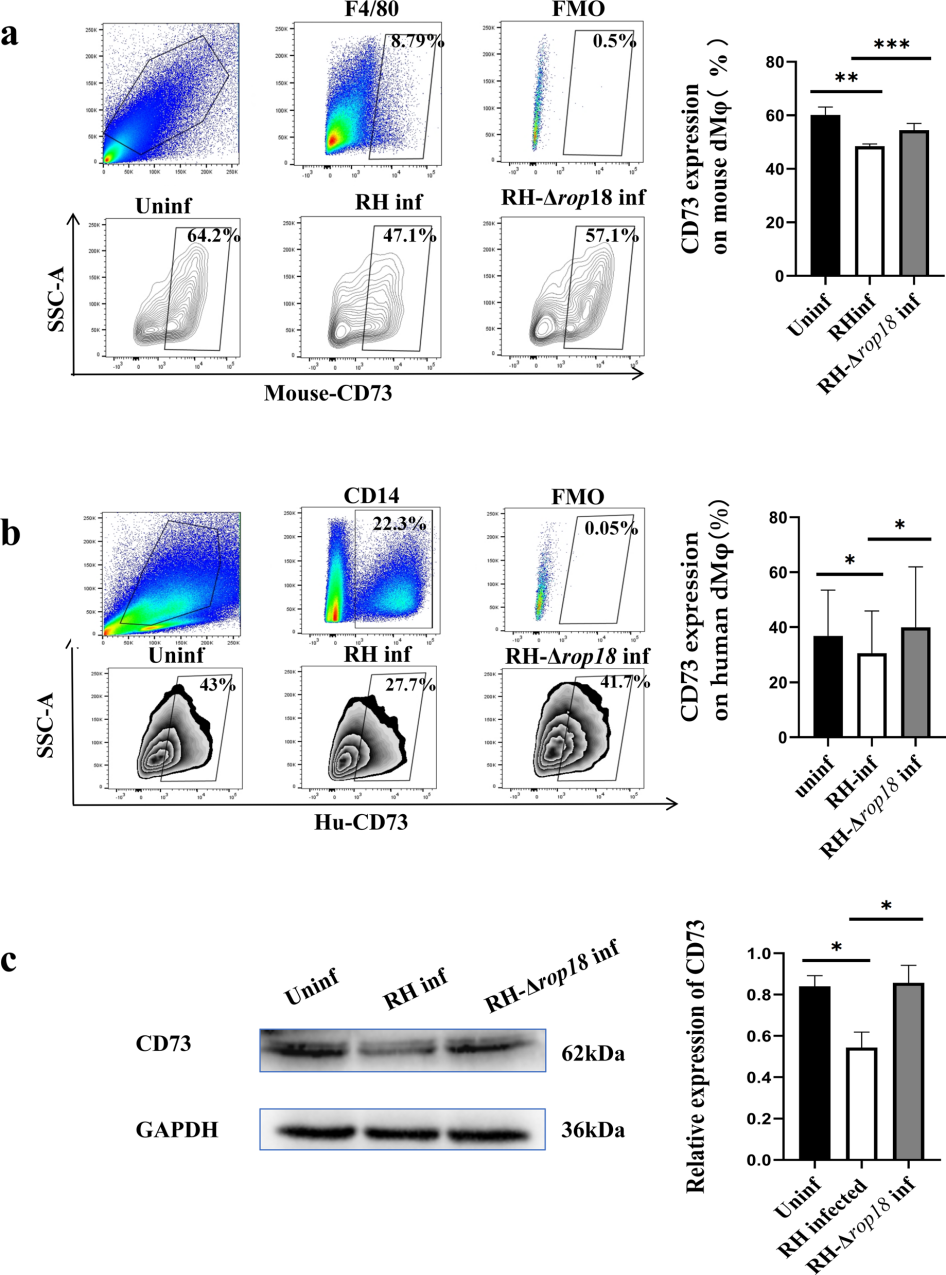
Expression levels of CD73 on dMφ in the uninfected, RH-infected, and RH-Δ*rop18*-infected groups. (**a**), CD73 expression on mouse dMφ in the uninfected, RH-infected, and RH-Δ*rop18*-infected mice analyzed with flow cytometry. (**b**), CD73 expression on human dMφ in the uninfected, RH-infected, and RH-Δ*rop18*-infected mice analyzed with flow cytometry The mouse data were identified by unpaired *t*-test, and human data by paired *t*-test. Data are presented as mean ± SD, ^*^P < 0.05, ^**^P < 0.01, ^***^P < 0.001. (**c)**, CD73 expression on human dMφ detected with western blot. Data are presented as mean ± SD, ^*^P < 0.05 (paired *t*-test)

To explore how *T*. *gondii* infection during pregnancy affects CD73 expression on dMφ, we used flow cytometry to analyze CD73 expression levels in pregnant mice from the three groups (uninfected, RH-infected, and RH-Δ*rop18*-infected group). The results showed that CD73 expression on dMφ was significantly lower in the RH-infected group than that in the uninfected group (*P* < 0.01). Meanwhile, CD73 expression was significantly higher in the RH-Δ*rop18*-infected group compared with that in the RH-infected group (*P* < 0.001) (Fig. 2a). Similarly, CD73 expression on human dMφ showed comparable results to those obtained in mice (*P* < 0.05) (Fig. 2b, c).

### Downregulation of CD73 expression on dMφ after *T*. *gondii* infection regulated through the LSD1/SNAIL1 pathway

**Fig. 3:**
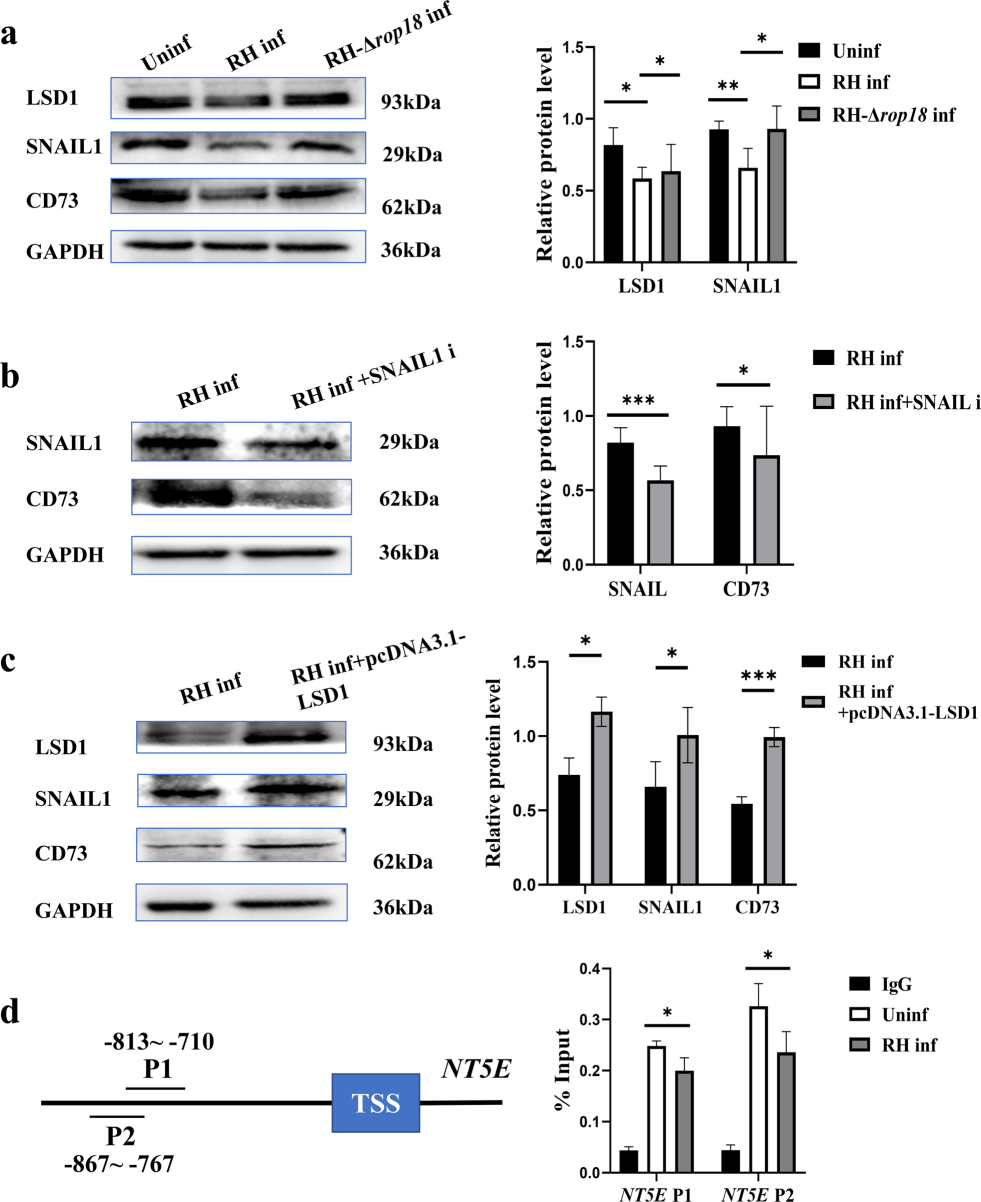
Downregulated expression of CD73 on dMφ via LSD1/SNAIL1 pathway during *T*. *gondii* infection. (**a**), LSD1 and SNAIL1 expression on purified human dMφ in the uninfected, RH-infected, and RH-Δ*rop18*-infected groups by western blot. (**b**), SNAIL1 and CD73 expression in the RH-infected and RH-infected + SNAIL1 inhibitor groups. (**c**), LSD1, SNAIL1, and CD73 expression in the RH-infected and RH-infected + pcDNA3.1-LSD1 groups. (**d**), The predicted binding site of SNAIL on the promoter regions of *NT5E* and CHIP-PCR results of SNAIL binding to *NT5E* promoter. ChIP assays were conducted with anti- SNAIL antibody, followed by qRT-PCR. All data are presented as mean ± SD, ^*^P < 0.05, ^**^P < 0.01, ^***^P < 0.001 (paired *t*-test)

Previous studies have demonstrated that SNAIL1 can directly bind to the proximal promoter of *NT5E*, resulting in upregulated CD73 expression [22]. And SNAIL1 has been observed to interact with LSD1 [23]. Therefore, we analyzed the expression levels of SNAIL1, its regulatory factor LSD1, and CD73 on dMφ to investigate the molecular mechanism underlying the downregulation of CD73 expression on dMφ following *T*. *gondii* infection. The results showed that the expression levels of LSD1 (*P* < 0.05), SNAIL1 (*P* < 0.01), and CD73 in human dMφ decreased in the RH-infected group compared with the uninfected group. However, they were all increased in the RH-Δ*rop18*-infected group compared with the RH-infected group (*P* < 0.05) (Fig. 3a). To further confirm the relationship of LSD1, SNAIL1, and CD73 during *T*. *gondii* infection, we used a SNAIL1 inhibitor and an overexpressed LSD1 plasmid in infected human dMφ and in induced THP-1 infected cells, respectively. The results revealed that CD73 expression decreased with the SNAIL1 inhibitor (*P* < 0.05) (Fig. 3b), while the expression levels of SNAIL1 (*P* < 0.05) and CD73 (*P* < 0.001) increased with LSD1 overexpression in induced THP-1 cells compared with those in the infected group (Fig. 3c). Additionally, to explore whether SNAIL1 could bind to the *NT5E* promoter during *T*. *gondii* infection, ChIP-qPCR was performed. The results showed that SNAIL1 could bind to the promoter regions (−813 ~ −710 bp, −867 ~ −767 bp) of *NT5E* under *T*. *gondii* infection conditions. We also found that *T*. *gondii* infection reduced the binding of SNAIL1 to *NT5E* promoter (*P* < 0.05) (Fig. 3d).

### ***CD73***^***−/−***^*** pregnant mice exhibit more severe adverse pregnancy outcomes after T. gondii infection than WT pregnant mice***

**Fig. 4:**
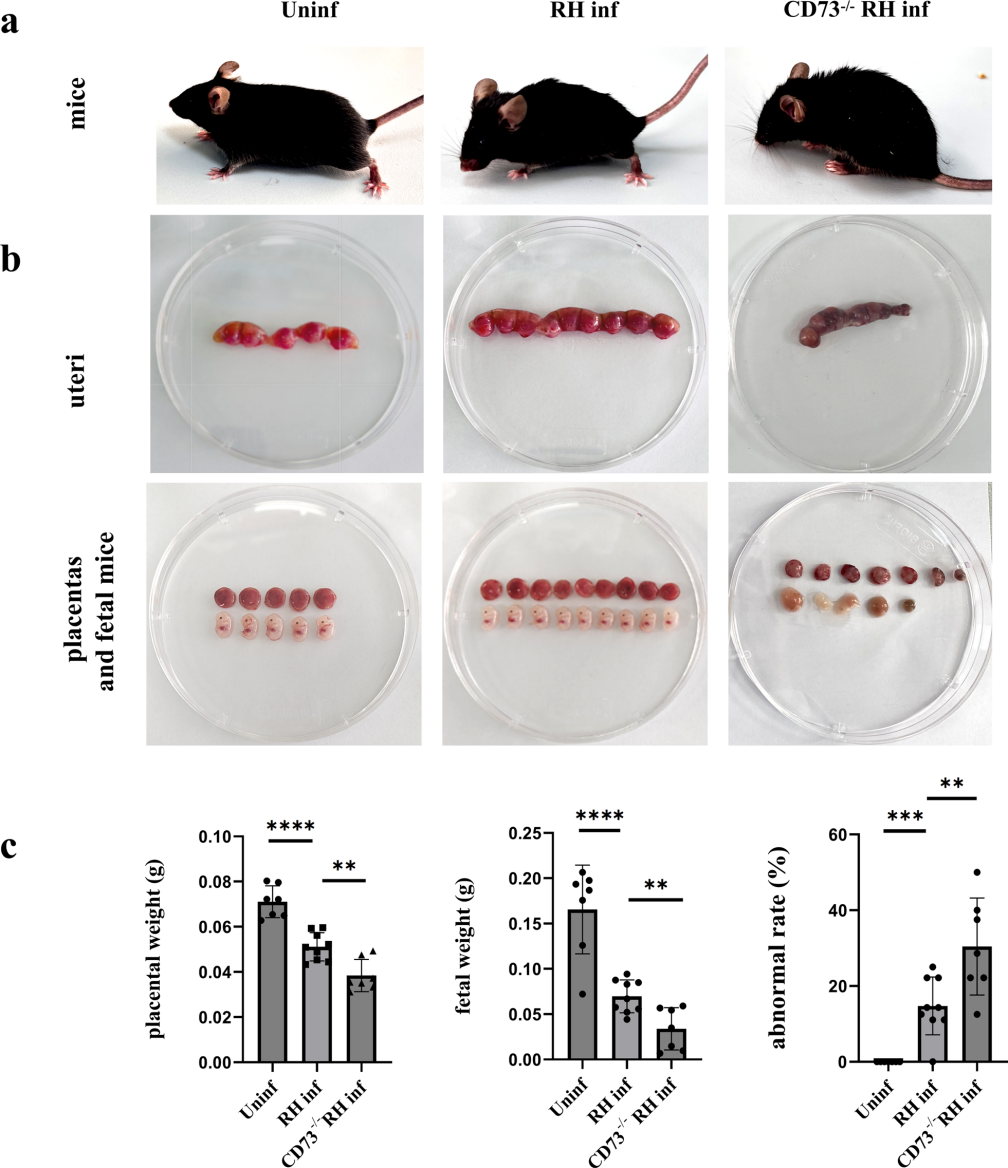
Pregnancy outcomes of the uninfected, infected, and CD73^−/−^infected mice. (**a**), Pregnant mice in the uninfected, infected, and CD73^−/−^infected groups. (**b**), Uteri, placentas, and fetuses in the uninfected, infected, and CD73^−/−^infected groups. (**c)**, Statistical analysis of average placental and fetal weights, and percentage of abnormal fetuses in the uninfected, infected, and CD73^−/−^infected groups. At least six pregnant mice in each group were identified by unpaired *t*-test. Data are presented as mean ± SD, ^**^P < 0.01, ^***^P < 0.001, ^****^P < 0.0001 (unpaired *t*-test)

In this study, we found that *T*. *gondii* infection significantly downregulates CD73 expression on dMφ. To investigate whether CD73 expression is related to adverse pregnancy outcomes induced by *T*. *gondii* infection, we established CD73^−/−^ infected pregnant mice. The observation of the pregnant mice showed that the CD73^−/−^ infected mice showed stagnant behavior, arched backs, erect hair, and drooping tails, while WT infected mice were slightly slow-moving, with slightly arched backs, and slightly raised hair and tails (Fig. 4a). Also, CD73^−/−^ infected pregnant mice exhibited more severe adverse pregnancy outcomes than the WT infected group. These mice exhibited more severe adverse pregnancy outcomes than the WT infected group. The uterus appeared smaller and shorter, with increased bleeding and necrosis. Placental bleeding showed increased severity, and nearly no normal fetuses were observed (Fig. 4b). Additionally, the average placental (*P* < 0.01) and fetal weights (*P* < 0.01) were decreased in the CD73^−/−^infected group, and the rate of abnormal fetuses (*P* < 0.01) was significantly higher compared with the WT infected group (Fig. 4c).

### Reduced CD73 expression after *T*. *gondii* infection regulates human dMφ activation

**Fig. 5:**
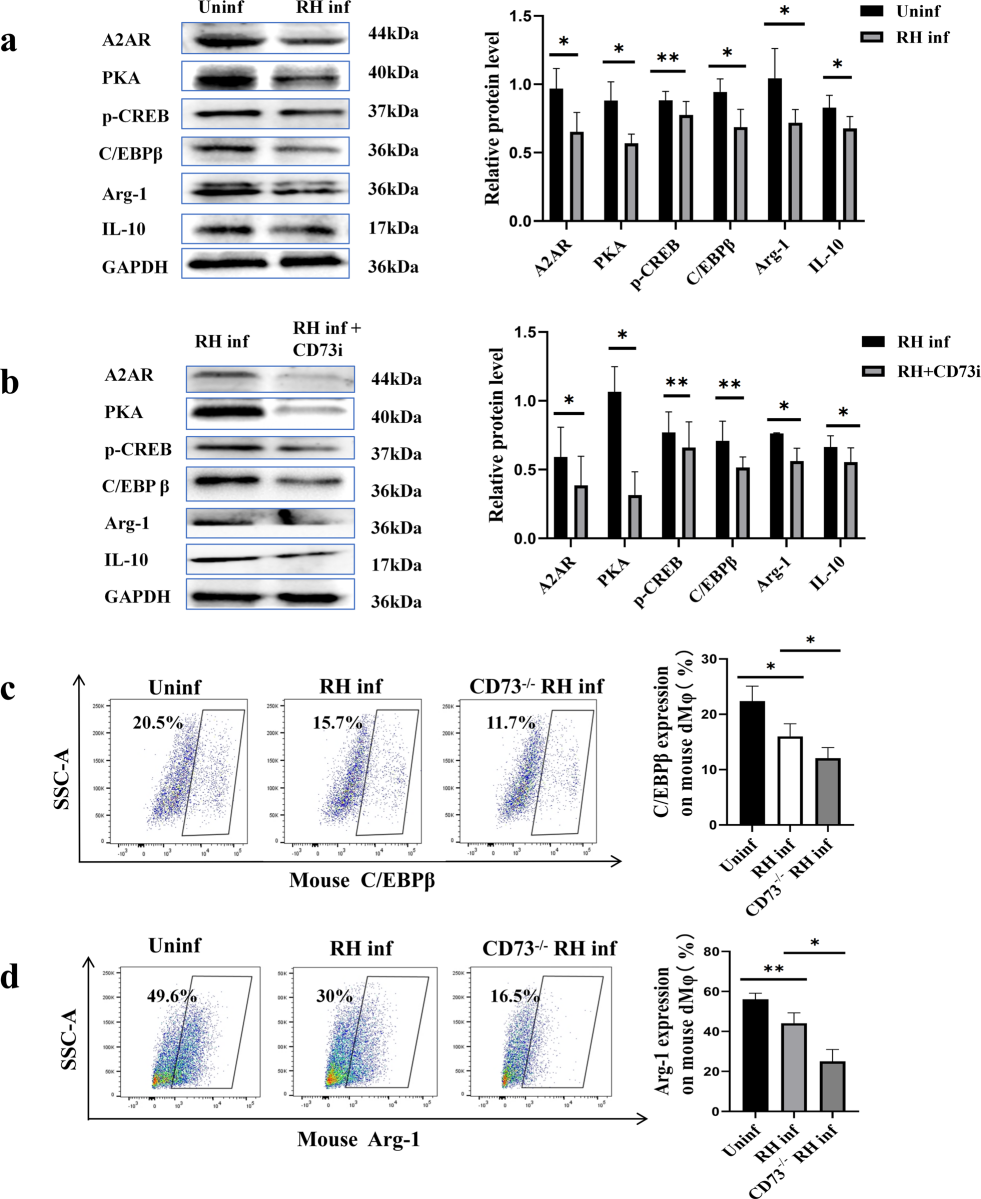
Decreased expression of A2AR, PKA, P-CREB, C/EBPβ, Arg-1, and IL-10 during *T*. *gondii* infection. (**a**), Expression of A2AR, PKA, p-CREB, C/EBPβ, Arg-1, and IL-10 in the uninfected and RH-infected human dMφ analyzed with western blot. (**b**), Expression of A2AR, PKA, p-CREB, C/EBPβ, Arg-1, and IL-10 in the infected and infected + CD73i human dMφ. (**c**), Expression of C/EBPβ in the uninfected, infected, and CD73^−/−^ infected mouse dMφ. (**d**), Arg-1 in the uninfected, infected, and CD73^−/−^ infected mouse dMφ. The human data were identified by paired *t*-test, and mouse data by unpaired *t*-test. All data are presented as mean ± SD, ^*^P < 0.05, ^**^P < 0.01

To investigate whether expression level of CD73 regulates the function of dMφ, we treated purified human dMφ with *T*. *gondii* infection and CD73i, then performed western blot analysis to examine the expression levels of immunosuppressive molecules Arg-1 and IL-10. In the condition of *T*. *gondii* infection, we found that the expression of Arg-1 (*P* < 0.05) and IL-10 (*P* < 0.05) were all decreased compared with those of the uninfected group (Fig. 5a). In addition, these levels were further declined when treated with CD73i in the infected group (*P* < 0.05) (Fig. 5b). In vivo, flow cytometry revealed that the percentage of Arg-1 positive cells in mouse dMφ were decreased after infection (*P* < 0.01) and further reduced in dMφ from CD73^−/−^ infected pregnant mice (*P* < 0.05) (Fig. 5d).

### Reduced CD73 expression on dMφ regulates Arg-1 and IL-10 expression through the A2AR/PKA/p-CREB/C/EBPβ pathway

Next, we explored how CD73 regulates the expression levels of Arg-1 and IL-10 in dMφ during *T*. *gondii* infection. The primary function of CD73 is to convert extracellular adenosine triphosphate (ATP) to Ado [24]. Additionally, Ado signaling through A2AR, a receptor for Ado highly expressed in macrophages, effectively suppresses immune responses in inflammatory tissues [25]. Western blot results showed a markedly decreased expression of A2AR treated with CD73i following *T*. *gondii* infection (*P* < 0.05) (Fig. 5a, b).

**Fig. 6:**
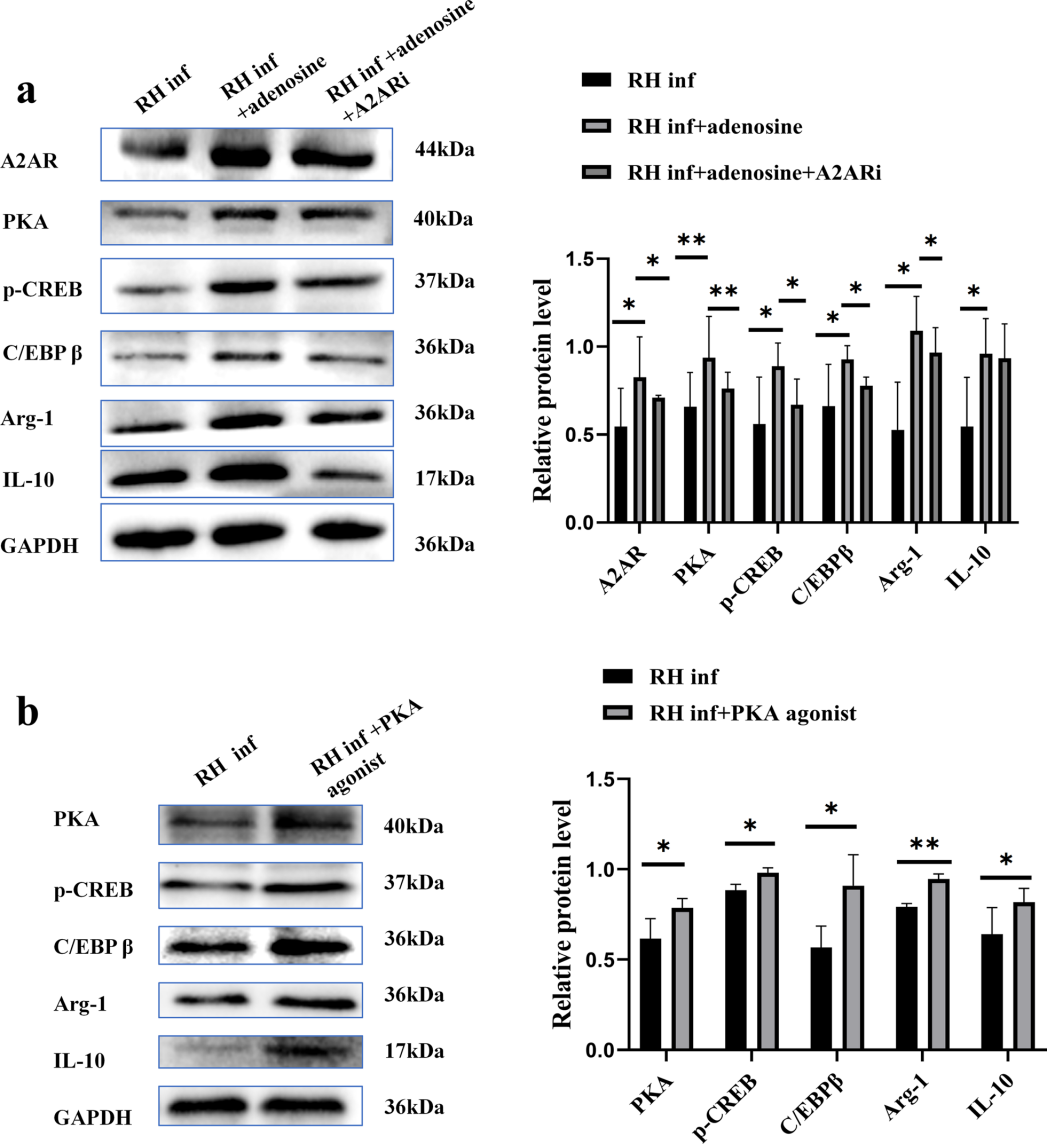
Reduced expression of CD73 in dMφ induced by *T*. *gondii* infection and its impact on Arg-1 and IL-10 expression through the A2AR/PKA/p-CREB/C/EBPβ pathway. (**a**), Western blot analysis of A2AR, PKA, p-CREB, C/EBPβ, Arg-1, and IL-10 expression levels in purified human dMφ from infected, infected treated with Ado, and infected treated with Ado and A2AR inhibitor groups (^*^P < 0.05, ^**^P < 0.01, via paired *t*-test). Data are presented as mean ± SD. (**b**), Western blot analysis of p-CREB, C/EBPβ, Arg-1, and IL-10 expression levels in human dMφ from infected and infected treated with PKA agonist groups (^*^P < 0.05, ^**^P < 0.01, paired *t*-test). Data are presented as mean ± SD

Studies have shown that the cAMP/ PKA/ CREB signaling pathway is positively regulated by CD73 and Ado [26]. Phosphorylated-CREB (p-CREB) promotes the transcription of C/EBPβ [27], which then enhances the expression of Arg-1 and IL-10 [28]. Therefore, exogenous Ado, A2AR inhibitor and PKA agonist were used to determine whether the expression level of CD73 regulates cAMP/PKA/CREB signaling pathway and C/EBPβ activity in dMφ during *T*. *gondii* infection. Reduced expression of PKA (*P* < 0.05), p-CREB (*P* < 0.01) and C/EBPβ (*P* < 0.05) were found in *T*. *gondii* infected dMφ and even lower in CD73i with *T*. *gondii* infected dMφ (Fig. 5a, b). Flow cytometry revealed that the percentage of C/EBPβ positive cells in mouse dMφ was decreased after infection and further reduced in dMφ from CD73^−/−^ infected mice (*P* < 0.05) (Fig. 5c). Supplementing exogenous Ado following *T*. *gondii* infection increased the expression levels of A2AR (*P* < 0.05), PKA (*P* < 0.01), p-CREB (*P* < 0.05), C/EBPβ (*P* < 0.05), Arg-1 (*P* < 0.05), and IL-10 (*P* < 0.05) in human dMφ compared with those in the group infected with *T*. *gondii* alone. However, when an A2AR inhibitor was added, the expression levels of these molecules were significantly downregulated (Fig. 6a). Furthermore, after infection and the addition of a PKA activator, the expression levels of p-CREB (*P* < 0.05), C/EBPβ (*P* < 0.05), Arg-1 (*P* < 0.01), and IL-10 (*P* < 0.05) were significantly upregulated in human dMφ compared with those of the group infected with *T*. *gondii* alone (Fig. 6b).

**Fig. 7:**
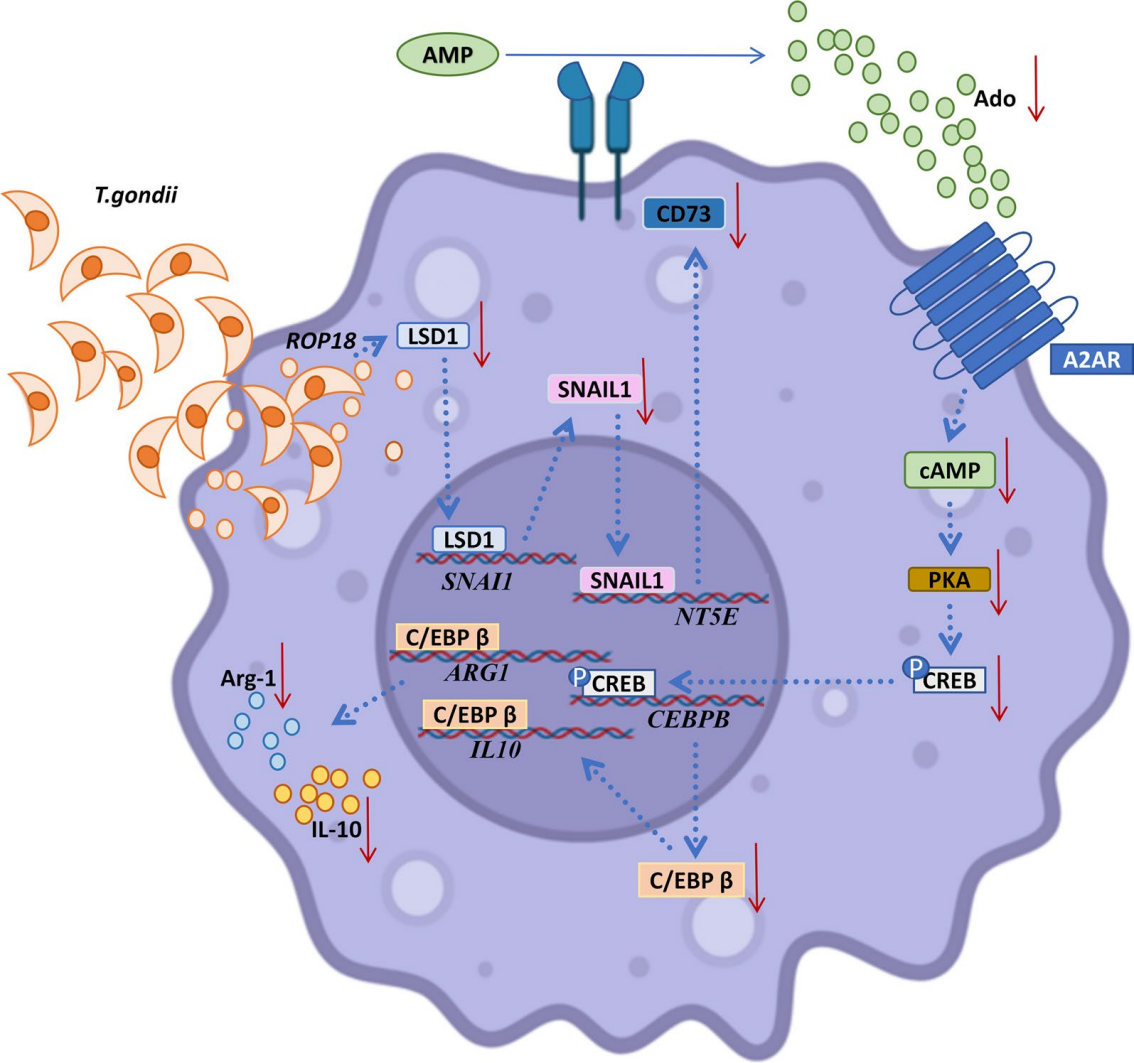
Mechanism of CD73 downregulated expression induced by *Tg*ROP18 and its consequences on dMφ dysfunction. *Tg*ROP18 downregulates CD73 expression on dMφ through the LSD1/SNAIL1 signaling pathway. The reduction in CD73 expression subsequent to *T*. *gondii* infection leads to reduced expression levels of Arg-1 and IL-10 through the Ado/A2AR/PKA/p-CREB/C/EBPβ signaling pathway, ultimately resulting in the dysfunctional maternal–fetal tolerance of dMφ

These results suggest that *T*. *gondii* invades to dMφ and then *Tg*ROP18, a high virulence factor, inhibits dMφ activation by downregulating the expression level of CD73, which result in the decrease of Arg-1 and IL-10 expression, eventually contributing to the maternal–fetal tolerance dysfunction of dMφ (Fig. 7).

## Discussion

At the maternal–fetal interface, maintaining an appropriate immune microenvironment is crucial for successful pregnancy [3]. This microenvironment relies on specific immune cells and their cytokines, which aid in embryo implantation, endometrial decidualization, and maternal tolerance of the fetus [8, 29]. *T*. *gondii*, an important opportunistic protozoan, can disrupt the immune microenvironment at the maternal–fetal interface during early pregnancy, leading to adverse pregnancy outcomes [6]. Among the immune cells involved, dMφ are particularly significant as they constitute the second largest population at this interface [8]. CD73 is a newly recognized immune suppressive molecule prominently expressed in tumor cells and in infiltrating immune cells within the tumor microenvironment [30]. Its primarily functions in conjunction with CD39 to convert extracellular ATP into Ado. Upon binding to Ado receptors, Ado inhibits immune responses and mitigates inflammation. Additionally, Ado influences the maturation, differentiation, and function of various immune cells [31]. At the maternal–fetal interface, CD73 is predominantly expressed in antigen-presenting cells, such as macrophages and dendritic cells. Clinical data indicate that patients with URSA exhibit significantly lower levels of CD73 in decidual and placental tissues, suggesting a potential involvement of CD73 in miscarriage occurrence [14]. However, whether the change of CD73 expression level on dMφ after *T*. *gondii* infection and whether this change was linked to adverse pregnancy outcomes require further investigation. In this study, CD73^−/−^ infected pregnant mouse models were established, and the pregnancy outcomes were observed. The findings revealed that adverse pregnancy outcomes were more severe in *T*. *gondii*–infected CD73^−/−^ pregnant mice compared with *T*. *gondii*–infected WT mice, indicating that alteration in CD73 expression level may play a crucial role in abnormal pregnancy outcomes induced by *T*. *gondii* infection. Furthermore, purified human dMφ infected with the *T*. *gondii* RH strain showed a significant decrease in CD73 expression. Additionally, our animal model of *T*. *gondii*–induced adverse pregnancy outcomes demonstrated a notable decrease in CD73^+^ dMφ in pregnant mice following infection. These combined in vitro and in vivo results indicated that CD73 expression on dMφ was significantly downregulated by *T*. *gondii* infection.

The structure of *T*. *gondii* is highly complex and growing evidence indicates that its virulence factors play crucial roles in invading host cells and regulating host immune response [32]. Among these factors, ROP18 stands out for its ability to suppress both innate and adaptive immune responses, highlighting its significance as a key virulence factor during *T*. *gondii* infection [19]. However, the impact of *Tg*ROP18 on abnormal pregnancy outcomes has not been well-documented. Thus, we established *T*. *gondii* RH- or RH-∆*rop18*-infected pregnant mouse models to explore the effect of *Tg*ROP18 on pregnancy outcomes. The findings revealed that pregnant mice infected with RH-*∆rop18* were exhibited better pregnancy outcomes than those of mice infected with *T*. *gondii* RH, suggesting a critical role of *Tg*ROP18 in adverse pregnancy outcomes. Additionally, flow cytometry and western blot results showed that the expression level of CD73 on human dMφ significantly increased in the RH-Δ*rop18*-infected group compared with that in the RH-infected group. In vivo experiments also confirmed a significant increase in the percentage of CD73^+^ dMφ in the RH-∆*rop18*-infected group compared with the RH-infected group. These experiments collectively suggest that *Tg*ROP18 plays a pivotal role in downregulating CD73 expression on dMφ.

The nuclear transcription factor SNAIL1 belongs to the zinc finger protein transcription factor family and functions in DNA binding and transcriptional regulation. It is overexpressed in various malignant tumors and positively correlates with tumor invasion [33–37]. Studies on human preeclampsia and a rat model of salt-induced preeclampsia have reported significantly reduced SNAIL1 levels in placental extracts[35]. Additionally, it has been identified a significant positive correlation between CD73 expression and SNAIL1, with SNAIL1 directly binding to the *NT5E* promoter [22]. However, whether SNAIL1 still regulates CD73 expression on dMφ during *T*. *gondii* infection remains uncertain. In this study, purified human dMφ were infected with either the *T*. *gondii* RH strain or the RH-Δ*rop18* strain. Western blot analysis revealed that SNAIL1 expression in human dMφ was significantly decreased following *T*. *gondii* RH infection, while it was increased in the RH-Δ*rop18*-infected group compared with the RH-infected group. Furthermore, we observed that the expression level of CD73 on dMφ was significantly downregulated upon treatment with a SNAIL1 inhibitor, suggesting that SNAIL1 may regulate CD73 expression during *T*. *gondii* infection. ChIP-qPCR results confirmed that SNAIL1 binds to *NT5E* promoter regions (−813 ~ −710 bp, −867 ~ −767 bp). Furthermore, our results demonstrated that *T*. *gondii* infection led to a reduction in the enrichment of SNAIL1 at the *NT5E* promoter region.

LSD1, the first discovered histone/lysine demethylase, is highly expressed in various cancers, including bladder and lung cancers, and is typically associated with advanced cancer stages and poor prognosis [38–40]. It has been illustrated that LSD1 interacts with SNAIL1, with the amine oxidase domain of LSD1 crucially interacting with the SNAG domain of SNAIL1 [22]. However, whether LSD1 regulates CD73 expression on dMφ during *T*. *gondii* infection remains unclear. Our results showed that LSD1 expression in human dMφ was reduced in the RH-infected group compared with that of the uninfected group, while it increased in the RH-Δ*rop18* infection group compared with that of the RH-infected group. Additionally, we designed a eukaryotic overexpression plasmid pcDNA3.1-LSD1, which was transiently transfected into induced THP-1 cells. Results showed that overexpression of LSD1 followed by infection with the RH strain could significantly increase the expression levels of SNAIL1 and CD73. This suggests that LSD1 acts as an upstream regulatory molecule of SNAIL1 and reveals *Tg*ROP18-reduced CD73 expression on dMφ through the LSD1/SNAIL1 signaling pathway.

In our previous studies, the results showed that decreased levels of functional molecules, such as Arg-1 and IL-10, in dMφ following *T*. *gondii* infection could disrupted its tolerance function [9–11]. However, it remained uncertain whether the downregulated expression of CD73 after *T*. *gondii* infection could regulate the expression levels of Arg-1 and IL-10, thereby contributing to dMφ dysfunction*.* To explore it, human dMφ infected with *T*. *gondii* were treated with CD73i. The results demonstrated that significant downregulation of Arg-1 and IL-10 expression in human dMφ compared with the *T*. *gondii*-infected group, confirming that CD73 downregulated expression on dMφ can lead to decreased expression of Arg-1 and IL-10. Additionally, the percentage of Arg-1 positive cells in CD73^−/−^ infected pregnant mice was significantly lower than those in WT infected pregnant mice. Jointly, these findings suggest that CD73 downregulation on dMφ after *T*. *gondii* infection further diminishes the expression of functional molecules Arg-1 and IL-10. However, the detailed molecular mechanism underlying these observations requires further investigation.

It has been confirmed that the primary function of CD73 is to convert extracellular ATP into Ado. To investigate whether the downregulated expression of CD73 on dMφ after *T*. *gondii* infection affects the expression levels of Arg-1 and IL-10, exogenous Ado was added to infected human dMφ. The results showed that the expression levels of Arg-1 and IL-10 in human infected dMφ significantly increased with the addition of Ado. This finding indicates that the downregulated expression of CD73 on dMφ following *T*. *gondii* infection indeed regulates the expression levels of Arg-1 and IL-10 by affecting Ado production.

Extracellular Ado produced by CD73 has been found to bind to Ado receptors (A1R, A2AR, A2BR, and A3R) to inhibit immune responses, reduce inflammation, and regulate the maturation, differentiation and function of various immune cells [25, 41, 42]. Among these receptors, A2AR is a typical GPCR with a high affinity for Ado, expressed in many immune cells, such as macrophages, regulatory T cells, and cytotoxic T cells [43]. To investigate whether the downregulated expression of CD73 on dMφ after *T*. *gondii* infection leads to decreased Ado binding to A2AR, exogenous Ado or exogenous Ado plus an A2AR inhibitor were added to human infected dMφ. The results showed that the expression levels of Arg-1 and IL-10 in human infected dMφ were significantly decreased when treated with exogenous Ado plus an A2AR inhibitor, while they increased with Ado alone. This indicates that the decrease in CD73 expression on dMφ after infection regulates the expression of Arg-1 and IL-10 by influencing the binding of Ado to A2AR.

It has been reported that extracellular Ado produced by CD73 could regulate the activity of AC, which converts intracellular ATP to cAMP [41]. CD73 and Ado can positively regulate the cAMP/PKA/CREB signaling pathway [26]. Studies indicate that CREB is a transcriptional regulator of C/EBPβ in gastric cancer cells, with p-CREB significantly enriching the C/EBPβ promoter region, thus promoting C/EBPβ transcription and activating the CREB-C/EBPβ cascade [27]. C/EBPβ, in turn, can bind to the promoter regions of Arg-1 and IL-10, promoting their expression [26]. However, it was unclear whether the downregulated expression of CD73 after *T*. *gondii* infection leads to decreased levels of functional molecules Arg-1 and IL-10 through the PKA/p-CREB/C/EBPβ pathway. In this study, we found a significant decrease in PKA, p-CREB, and C/EBPβ levels in purified human dMφ following *T*. *gondii* infection. Similarly, in vivo experiments showed a significant decrease in the percentage of C/EBPβ^+^ dMφ in pregnant mice after infection. Additionally, PKA, p-CREB, and C/EBPβ expression levels were further downregulated in human infected dMφ treated with CD73i. We observed that the percentage of C/EBPβ^+^ cells in CD73^−/−^ infected pregnant mice was significantly lower than in WT infected mice. Furthermore, adding a PKA agonist to human infected dMφ significantly increased the expression levels of p-CREB, C/EBPβ, Arg-1, and IL-10. Our results demonstrated that the downregulation of CD73 expression after *T*. *gondii* infection affects the expression of Arg-1 and IL-10 through the Ado/A2AR/PKA/p-CREB/C/EBPβ signaling pathway.

## Conclusions

In summary, *Tg*ROP18 could downregulate CD73 expression on dMφ through the LSD1/SNAIL1 signaling pathway, then the decrease of CD73 expression reduced the expression levels of Arg-1 and IL-10 through the Ado/A2AR/PKA/p-CREB/C/EBPβ signaling pathway, ultimately resulting in maternal–fetal tolerance dysfunction of dMφ, contributing to adverse pregnancy outcomes.

## Data Availability

No datasets were generated or analyzed during the current study.

## Abbreviations

dMφ: Decidual macrophage
FBS: Fetal bovine serum
SNAIL1: Snail family transcriptional repressor 1
LSD1: Lysine-specific histone demethylase1
ATP: Adenosine triphosphate
A2AR: Adenosine A2a receptor
cAMP: Cyclic adenosine monophosphate
PKA: Protein Kinase A
CREB: cAMP-response element binding protein
C/EBPβ: CCAAT enhancer binding protein β
Arg-1: Arginase 1
IL-10: Interleukin 10
